# Supplementary material for: Evolution of Therapeutic Patient Education: A Systematic Scoping Review and Scientometric Analysis
Source: Int J Environ Res Public Health. 2022 May 18;19(10):6128. doi: 10.3390/ijerph19106128 (PMC9140728; doi:10.3390/ijerph19106128)
Supplement: Supplementary file 1 [file ijerph-19-06128-s001.zip › ijerph-1651074-supplementary.pdf]

Supplementary Material

Supplementary Figure S1: Disciplines of research central in patient education research

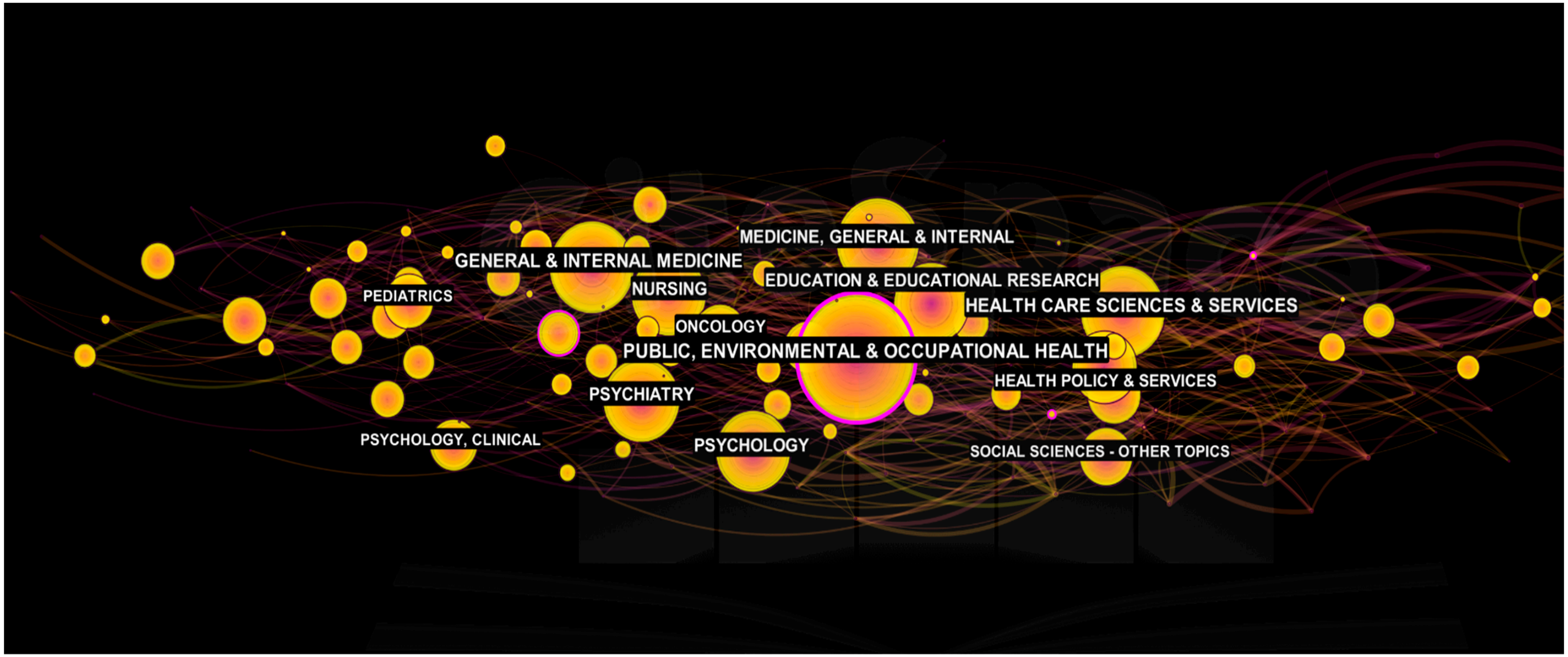

Supplementary Figure S2: Collaborative network of countries in patient education research

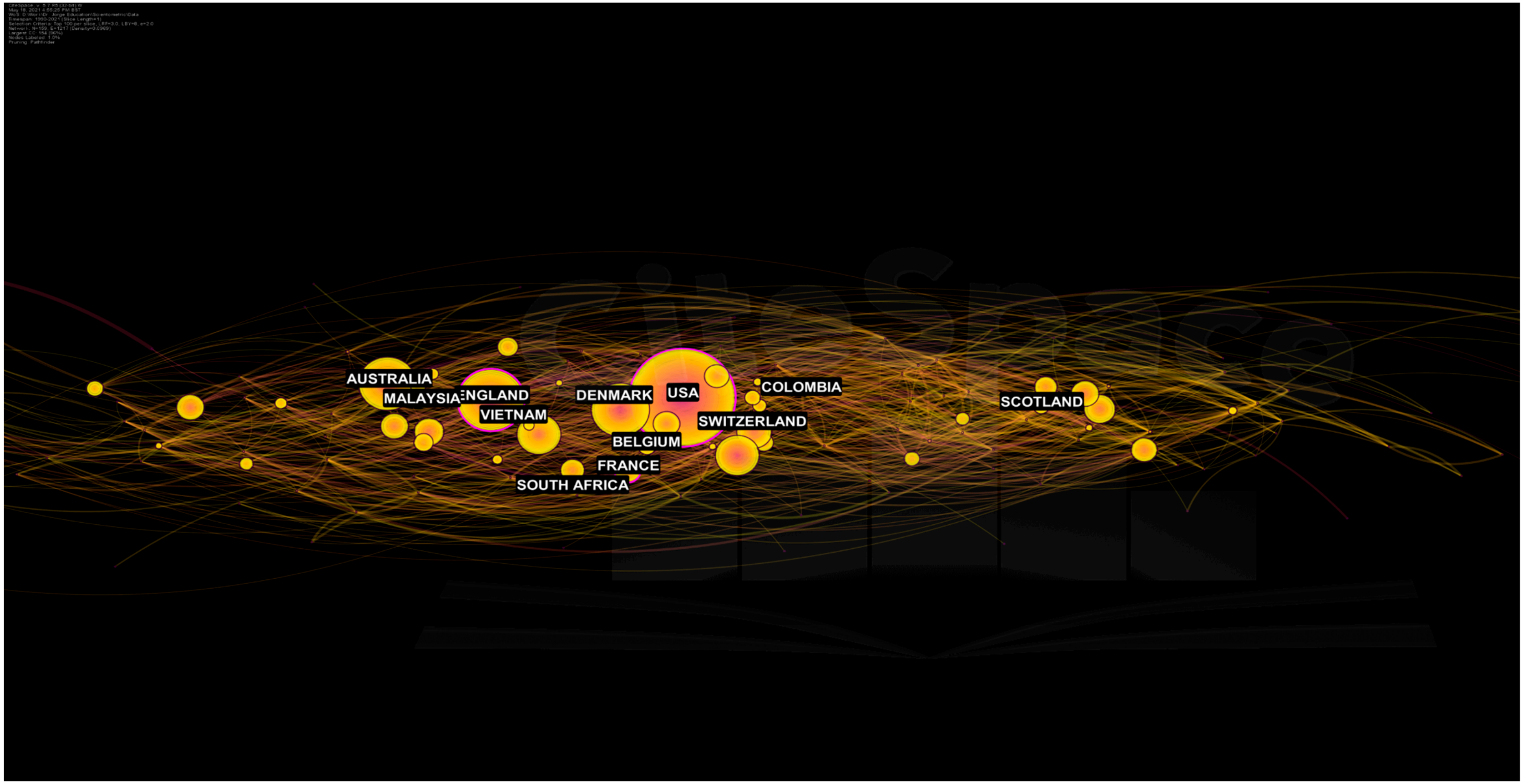

Supplementary Figure S3: Top institutions contributing to patient education research

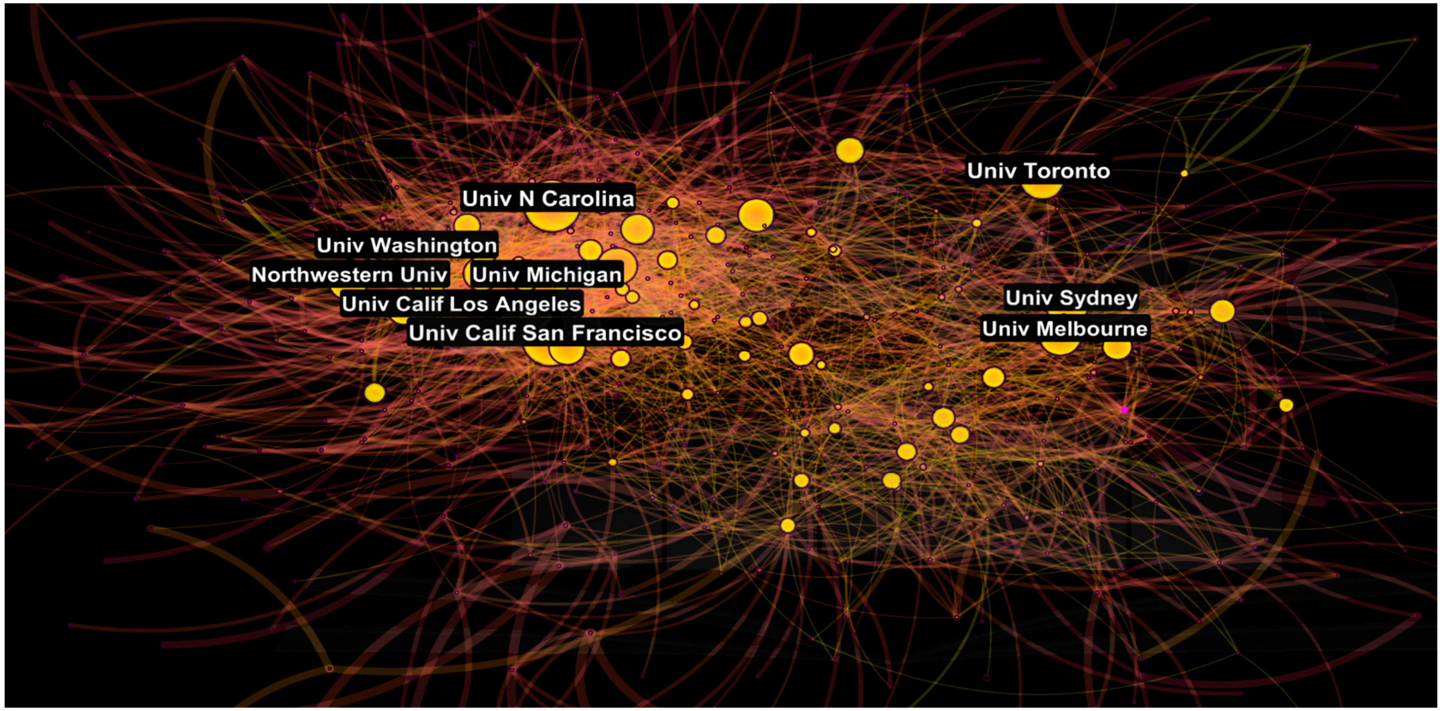

Supplementary Figure S4: Keywords corresponding to central topics in patient education research

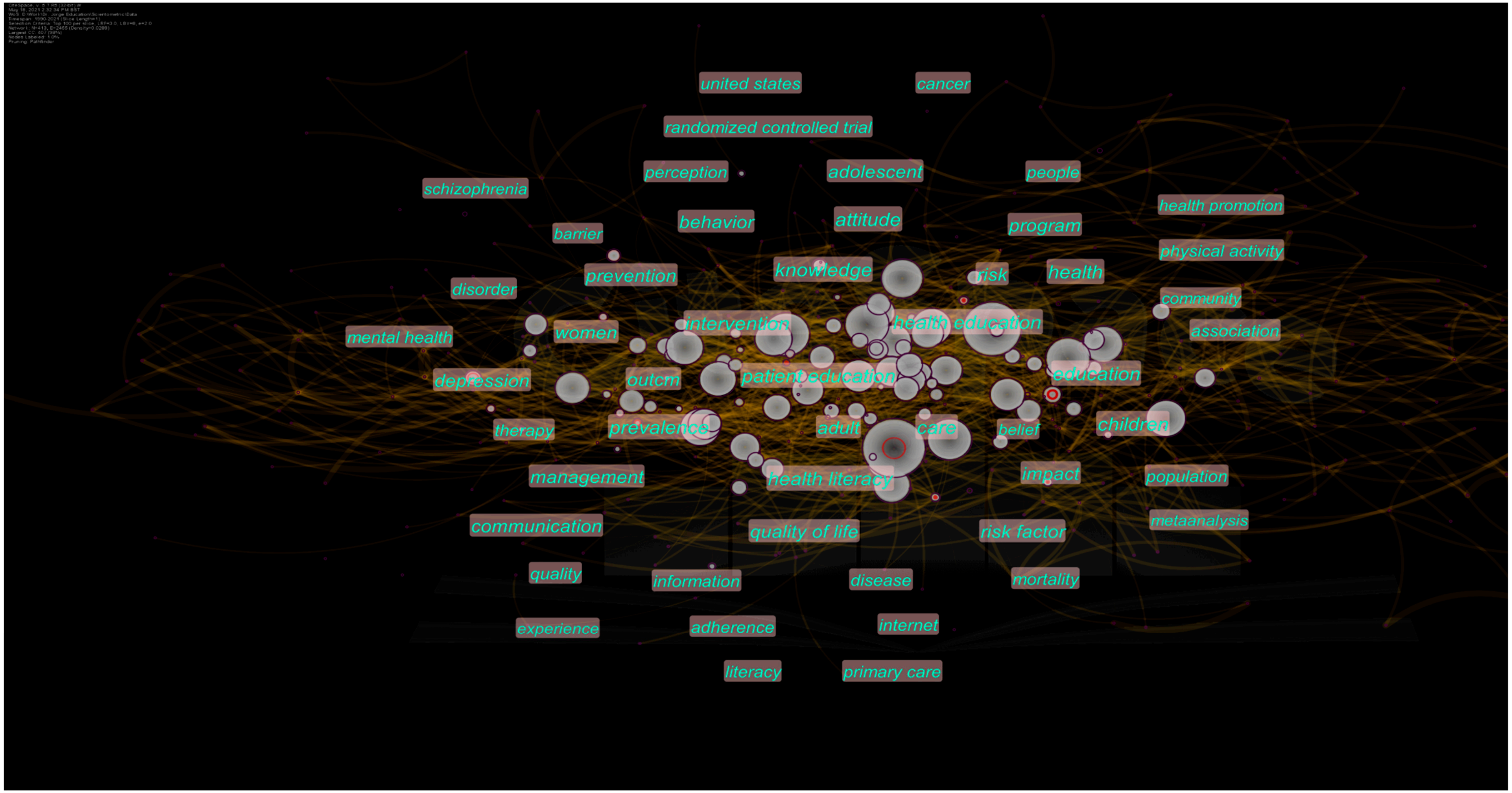

Supplementary Figure S5: Top research articles published from 1985 to 1996

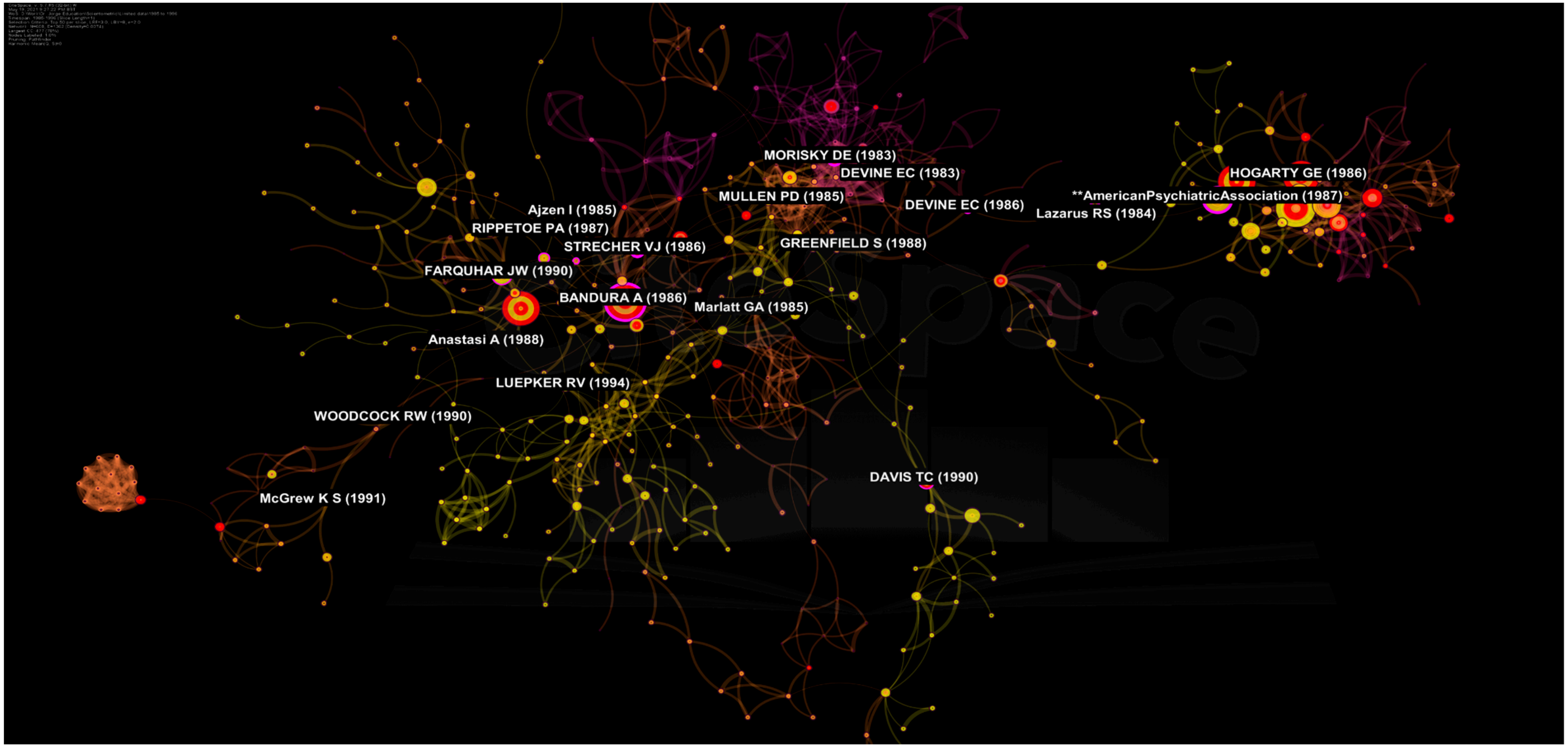

Supplementary Figure S6: Top articles in patient education research from 1997 to 2006

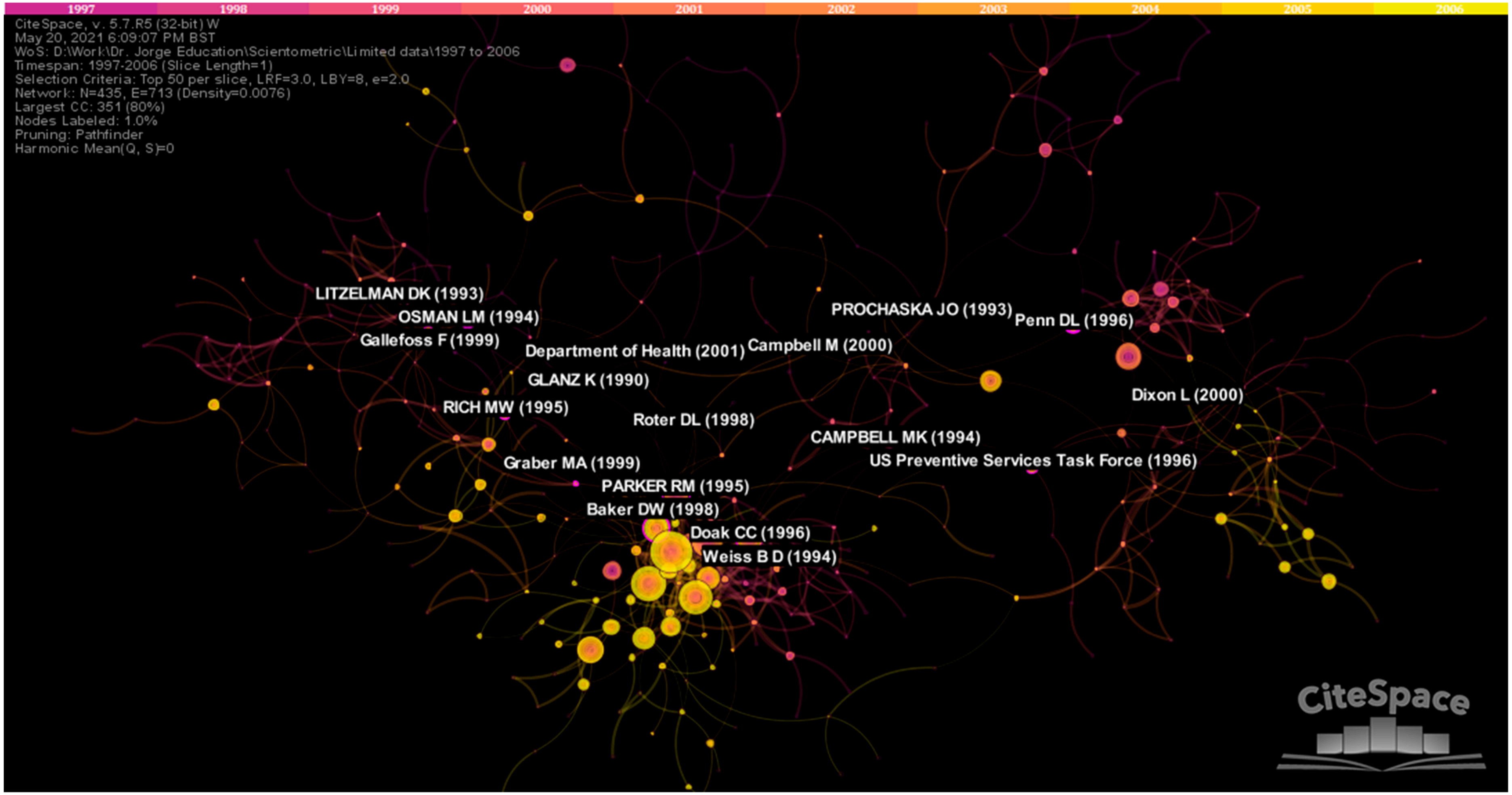

Supplementary Figure S7: Top articles published from 2007 to 2011

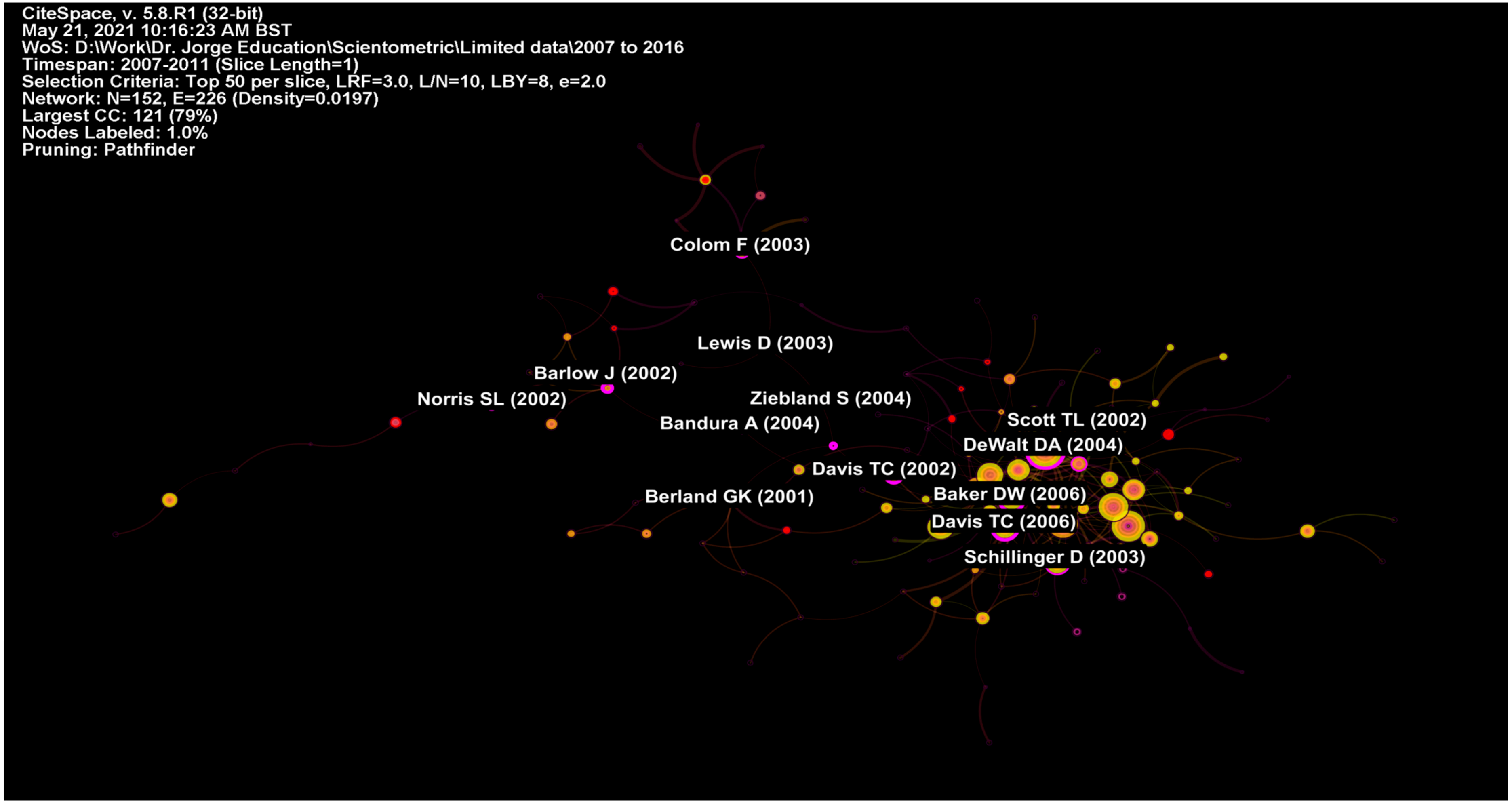

Supplementary Figure S8: Top articles published from 2012 to 2016

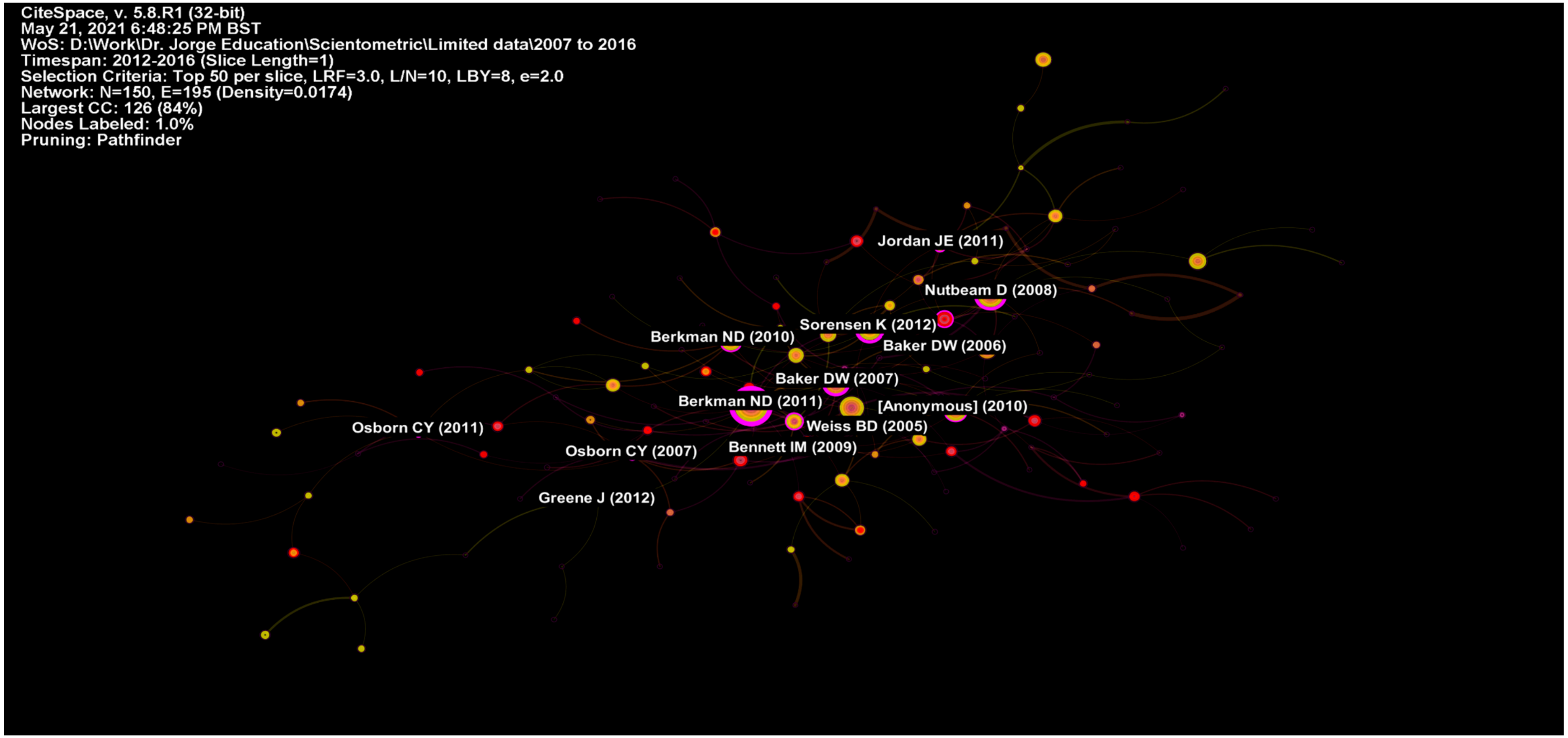

Supplementary Figure S9: Top articles published from 2017 to 2021

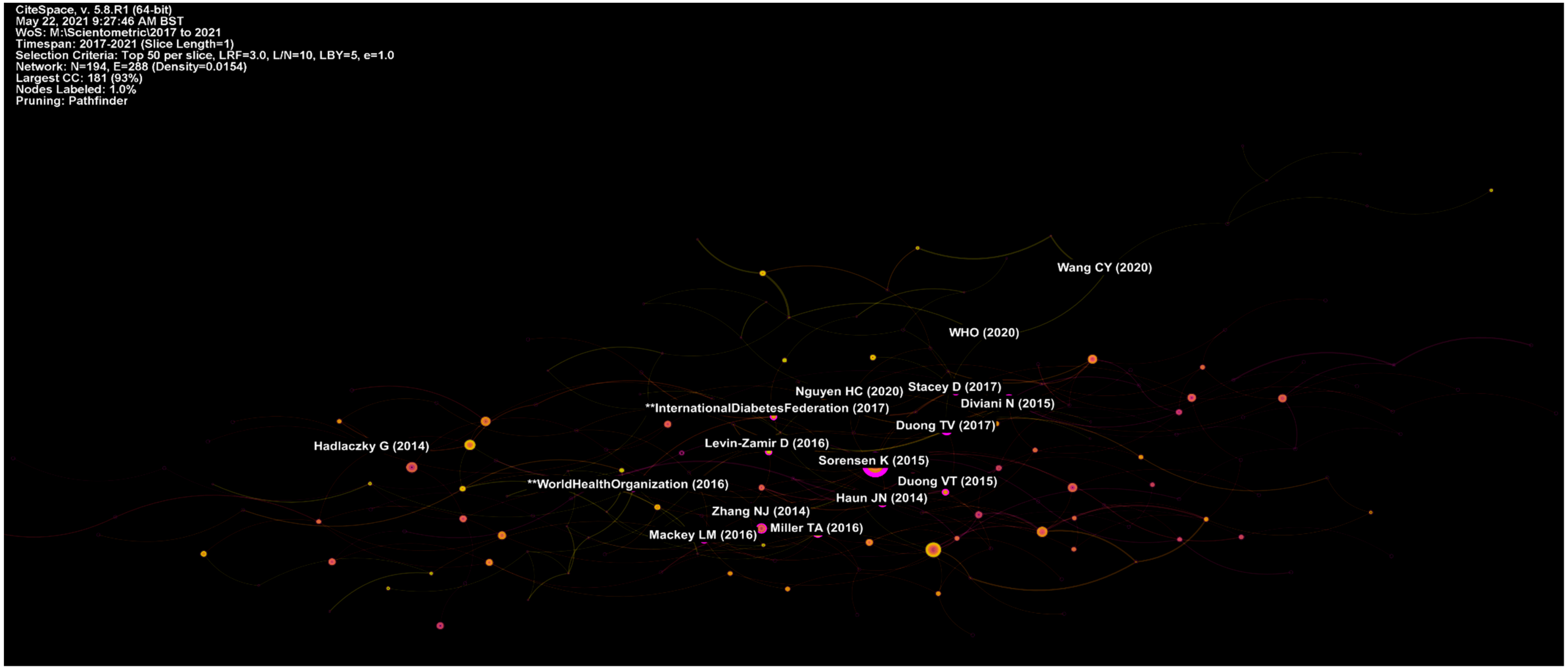

Supplementary Table S1: Top cited keywords in patient education research (>300 citations)

Theme	Keywords
**Patient education terms**	Literacy, psychoeducation,
**Disease/Disorder**	Depression, physical activity, cancer, primary care, mental health, schizophrenia, obesity, breast cancer, anxiety, exercise, infection, pregnancy, HIV, mental health literacy, hypertension, oral health, diabetes
**Study designs**	Implementation programme, RCT, association, barrier, experience, meta-analysis, validation, efficacy, epidemiology, model, trial, questionnaire, service, predictor, validity, qualitative research
**Scope**	Behavior, communication, information, internet, perception, belief, health promotion, public health, scale development, self-management, guideline, support
**Outcomes**	Knowledge, education, prevalence, attitude, impact, risk, management, prevention, quality of life, risk factor, adherence, mortality, symptom, diagnosis, health knowledge, awareness, illness, stigma, decision making
**Populations**	Children, adolescent, women, adult, Unites States, community, older adult, disparity, patient, student, physician, parent

Supplementary Table S2: Top funders contributing to the field of patient education

**Funding Agencies**	**Frequency**	**% of 54533**
UNITED STATES DEPARTMENT OF HEALTH HUMAN SERVICES	6881	12.618
NATIONAL INSTITUTES OF HEALTH NIH USA	6139	11.257
NIH NATIONAL CANCER INSTITUTE NCI	1196	2.193
EUROPEAN COMMISSION	1154	2.116
NIH NATIONAL INSTITUTE OF MENTAL HEALTH NIMH	781	1.432
NIH NATIONAL HEART LUNG BLOOD INSTITUTE NHLBI	748	1.372
NIH NATIONAL CENTER FOR ADVANCING TRANSLATIONAL SCIENCES NCATS	700	1.284
NIH NATIONAL INSTITUTE OF DIABETES DIGESTIVE KIDNEY DISEASES NIDDK	591	1.084
NIH NATIONAL INSTITUTE ON AGING NIA	574	1.053
NATIONAL HEALTH AND MEDICAL RESEARCH COUNCIL OF AUSTRALIA	546	1.001
NIH EUNICE KENNEDY SHRIVER NATIONAL INSTITUTE OF CHILD HEALTH HUMAN DEVELOPMENT NICHD	539	0.988
NIH NATIONAL CENTER FOR RESEARCH RESOURCES NCRR	476	0.873
CANADIAN INSTITUTES OF HEALTH RESEARCH CIHR	468	0.858
AGENCY FOR HEALTHCARE RESEARCH QUALITY	456	0.836
NIH NATIONAL INSTITUTE OF NURSING RESEARCH NINR	411	0.754
UK RESEARCH INNOVATION UKRI	392	0.719
CENTERS FOR DISEASE CONTROL PREVENTION USA	370	0.678
NATIONAL NATURAL SCIENCE FOUNDATION OF CHINA NSFC	370	0.678
NIH NATIONAL INSTITUTE ON DRUG ABUSE NIDA	362	0.664
MEDICAL RESEARCH COUNCIL UK MRC	311	0.57
NIH NATIONAL INSTITUTE ON MINORITY HEALTH HEALTH DISPARITIES NIMHD	299	0.548
MINISTRY OF EDUCATION CULTURE SPORTS SCIENCE AND TECHNOLOGY JAPAN MEXT	289	0.53
NATIONAL INSTITUTE FOR HEALTH RESEARCH NIHR	269	0.493
UNITED STATES PUBLIC HEALTH SERVICE	264	0.484
JAPAN SOCIETY FOR THE PROMOTION OF SCIENCE	247	0.453

Supplementary Table S3: Top keywords presented as citation bursts

Keywords	Year	Strength	Begin	End	1990 - 2021
controlled trial	1990	**129.93**	1991	2009	
patient education	1990	**106.52**	1995	2005	
asthma	1990	**103.39**	1991	2013	
clinical trial	1990	**68.8**	1994	2011	
follow up	1990	**68.55**	1991	2013	
comprehension	1990	**68.04**	2010	2014	
relapse	1990	**66.57**	1991	2006	
health education	1990	**66.33**	1990	2000	
stress	1990	**62.87**	2019	2021	
caregiver	1990	**61.6**	2018	2021	
mellitus	1990	**59.89**	1991	2004	
perspective	1990	**59.28**	2017	2018	
reliability	1990	**56.4**	2018	2021	
general practice	1990	**56.4**	1991	2005	
blood pressure	1990	**53.05**	1991	2008	
burden	1990	**52.61**	2016	2017	
promotion	1990	**51.97**	1991	2005	
rheumatoid arthriti	1990	**48.38**	1992	2003	
skill	1990	**47.75**	2010	2013	
morbidity	1990	**47.54**	1991	2005	
stigma	1990	**47.39**	2016	2021	

medication	1990	**46.74**	2007	2011	
pattern	1990	**46.38**	1991	2007	
smoking cessation	1990	**45.33**	1994	2006	
nursing	1990	**45.2**	2018	2021	
mental health	1990	**44.63**	2019	2021	
qualitative research	1990	**42.43**	2018	2021	
compliance	1990	**42.29**	1991	2007	
trial	1990	**40.89**	1991	2008	
life	1990	**40.51**	2019	2021	
youth	1990	**40.17**	2019	2021	
expressed emotion	1990	**39.81**	1991	2003	
rehabilitation	1990	**39.48**	2011	2014	
oral health	1990	**39.24**	2014	2018	
disability	1990	**37.9**	1993	2006	
survival	1990	**37.13**	1991	2008	
patienteducation	1990	**35.98**	1991	2005	
chronic disease	1990	**35.04**	2015	2016	
validation	1990	**34.88**	2019	2021	
alcohol	1990	**34.6**	1991	2006	
diabete	1990	**31.4**	2015	2017	
arthriti	1990	**30.51**	1991	2002	
trend	1990	**30.32**	1992	2007	
functional health literacy	1990	**29.74**	2005	2007	

men	1990	**29.42**	2008	2011	
nurse	1990	**29.37**	2007	2009	
determinant	1990	**28.73**	2014	2015	
diabetes mellitus	1990	**28.53**	1995	2007	
social support	1990	**27.38**	2018	2021	
decision making	1990	**25.99**	2015	2018	
glycemic control	1990	**25.71**	2003	2009	
mental health literacy	1990	**24.88**	2018	2021	
cigarette smoking	1990	**23.95**	1992	1998	
satisfaction	1990	**23.27**	2018	2021	
maintenance chemotherapy	1990	**22.78**	1993	1998	
aftercare treatment	1990	**22.78**	1993	1998	
metaanalysis	1990	**22.27**	2017	2018	
system	1990	**21.58**	1992	2007	
drug	1990	**21.18**	1991	1998	
childhood	1990	**21.07**	1991	2003	
cardiovascular disease	1990	**20.76**	2005	2008	
coronary heart disease	1990	**20.72**	2005	2007	
gender	1990	**20.04**	2009	2011	
hiv infection	1990	**20.02**	1992	2000	
health information	1990	**19.95**	2001	2008	
african american	1990	**19.56**	2013	2014	
strategy	1990	**18.94**	2018	2019	

physician	1990	**18.85**	1992	2005	
pain	1990	**18.58**	2013	2014	
cholesterol	1990	**18.22**	1991	1997	
psychotherapy	1990	**17.6**	2004	2007	
mother	1990	**17.56**	1991	2006	
awareness	1990	**17.47**	2018	2019	
children	1990	**17.26**	1995	1998	
adjustment	1990	**17**	1993	2002	
schizophrenic patient	1990	**16.54**	1992	1999	
self-management	1990	**16.5**	2018	2021	
randomized trial	1990	**15.92**	2013	2014	
family psychoeducation	1990	**15.83**	1991	1996	
access	1990	**15.69**	2008	2009	
health behavior	1990	**15.39**	1991	2004	
smoking	1990	**15.08**	2009	2010	
aid	1990	**14.9**	2007	2008	
diet	1990	**14.76**	1991	1998	
school	1990	**14.26**	1992	2000	
transmission	1990	**14.08**	1991	2000	
need	1990	**13.91**	2001	2003	
randomized controlled trial	1990	**13.9**	2006	2007	
complication	1990	**13.81**	1991	2005	
illness	1990	**13.57**	2001	2006	

elderly patient	1990	**13.37**	1999	2003	
age	1990	**13.07**	2005	2006	
africa	1990	**13.01**	1991	1997	
project	1990	**12.95**	1995	2004	
myocardial infarction	1990	**12.93**	1992	1998	
participation	1990	**12.83**	2002	2007	
family	1990	**12.74**	2009	2010	
issue	1990	**12.57**	1995	2005	
medication adherence	1990	**12.54**	2019	2021	
mammography	1990	**12.54**	1991	2004	
prevention	1990	**12.39**	1994	1997	
nigeria	1990	**12.29**	1991	2003	
self efficacy	1990	**12.24**	1995	1998	
hypertension	1990	**12.17**	2011	2013	
human immunodeficiency virus	1990	**12.01**	1991	1995	
primary health care	1990	**11.88**	1992	2000	
surgery	1990	**11.51**	2005	2007	
relative	1990	**11.12**	1991	2003	
readability	1990	**10.89**	2017	2018	
cost	1990	**10.85**	2005	2007	
emergency	1990	**10.79**	1998	1999	
world wide web	1990	**10.73**	2000	2004	
developing country	1990	**10.4**	1994	2002	

therapy	1990	**9.99**	1996	2000	
health status	1990	**9.94**	1991	2005	
infant	1990	**9.89**	1991	1996	
medical care	1990	**9.76**	1991	1997	
medicine	1990	**9.41**	1999	2000	
heart disease	1990	**9.17**	1992	1997	
exposure	1990	**9.12**	1991	2004	
education program	1990	**8.85**	1997	2002	
empowerment	1990	**8.79**	1995	2001	
maintenance	1990	**8.73**	1992	1995	
sexually transmitted disease	1990	**8.53**	1991	2001	
social cla	1990	**8.46**	1991	1997	
dental cary	1990	**8.37**	1990	2002	
program	1990	**8.03**	1999	2000	
informed consent	1990	**8.01**	1996	1998	
infection	1990	**7.86**	2010	2011	
mass media	1990	**7.86**	1992	1997	
consumption	1990	**7.67**	1992	2003	
treatment	1990	**7.46**	1995	1996	
belief	1990	**7.27**	2009	2011	
coping	1990	**7.25**	1995	2003	
malignant melanoma	1990	**7.16**	1995	1999	
performance	1990	**7.15**	2001	2004	

reduction	1990	**7.06**	1991	1998	
guideline	1990	**7**	2012	2014	
psychoeducation	1990	**6.8**	2006	2007	
preference	1990	**6.79**	1995	2002	
meta analysis	1990	**6.69**	1995	1997	
smoker	1990	**6.63**	1992	2002	
diarrhea	1990	**6.59**	1991	1996	
general practitioner	1990	**5.98**	1994	1997	
self care	1990	**5.94**	1993	2003	
childhood asthma	1990	**5.82**	1992	1998	
cary	1990	**5.39**	1991	1994	
evaluation	1990	**5.36**	1992	1997	
hospitalization	1990	**4.76**	1991	1993	
mortality	1990	**4.72**	1994	1995	
schoolchildren	1990	**4.7**	1992	1995	
immunization	1990	**4.65**	1992	1996	
behavioral intervention	1990	**4.65**	1992	1996	
sexual behavior	1990	**4.55**	1991	1997	
symptom	1990	**4.49**	2002	2003	
schizophrenia	1990	**4.21**	1996	1998	
aftercare	1990	**4.1**	1993	1994	
adolescence	1990	**4.1**	1993	1994	
low birth weight	1990	**4.07**	1992	1994	

recommendation	1990	**3.88**	1992	1993	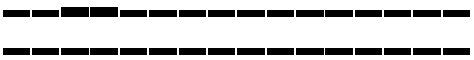

Supplementary Table S4: References yielding high centrality values in patient education research from 1922 to 2020.

**Pre 1985**
Farquhar J, Wood P, Breitrose H, Haskell W, Meyer A, Maccoby N, Alexander J, Brown JR B, Mcalister A, Nash J, Stern M. Community education for cardiovascular health. The Lancet. 1977 Jun 4;309(8023):1192-5.
Green LW, Figá-Talamanca I. Suggested designs for evaluation of patient education programs. Health Education Monographs. 1974 Feb;2(1):54-71.
Green LW. Toward cost-benefit evaluations of health education: some concepts, methods, and examples. Health Education Monographs. 1974 Mar;2(1_suppl):34-64.
**1985 to 1996**
Morisky DE, Levine DM, Green LW, Shapiro S, Russell RP, Smith CR. Five-year blood pressure control and mortality following health education for hypertensive patients. American journal of public health. 1983 Feb;73(2):153-62.
Devine EC, Cook TD. Clinical and cost-saving effects of psychoeducational interventions with surgical patients: A meta-analysis. Research in Nursing & Health. 1986 Jun;9(2):89-105.
**DSM**-III (1980). American Psychiatric Association. (1980). Diagnostic and Statistical Manual of Mental Disorders (**3rd ed**.)
Devine EC, Cook TD (1983). A meta-analytic analysis of effects of psychoeducational interventions on length of postsurgical hospital stay. Nursing Research Practice 32(5):267-74.
Folkman S, Lazarus RS. Stress, appraisal, and coping. New York: Springer Publishing Company; 1984.
Bandura A. Social foundations of thought and action. Englewood Cliffs, NJ. 1986;1986(23-28).
Strecher VJ, McEvoy DeVellis B, Becker MH, Rosenstock IM. The role of self-efficacy in achieving health behavior change. Health education quarterly. 1986 Mar;13(1):73-92.
Mullen PD, Green LW, Persinger GS. Clinical trials of patient education for chronic conditions: a comparative meta-analysis of intervention types. Preventive medicine. 1985 Nov 1;14(6):753-81.
Marlatt, G. A. (1985). Relapse prevention: Theoretical rationale and overview of the model. In G. A. Marlatt & J. R. Gordon (Eds.), Relapse prevention (1st ed., pp. 280–250). New York: Guilford Press.
Ajzen I. From intentions to actions: A theory of planned behavior. InAction control 1985 (pp. 11-39). Springer, Berlin, Heidelberg.
Farquhar JW, Fortmann SP, Flora JA, Taylor CB, Haskell WL, Williams PT, Maccoby N, Wood PD. Effects of communitywide education on cardiovascular disease risk factors: the Stanford Five-City Project. Jama. 1990 Jul 18;264(3):359-65.
Rippetoe PA, Rogers RW. Effects of components of protection-motivation theory on adaptive and maladaptive coping with a health threat. Journal of personality and social psychology. 1987 Mar;52(3):596.
Davis TC, Crouch MA, Wills G, Miller S, Abdehou DM. The gap between patient reading comprehension and the readability of patient education materials. J Fam Pract. 1990 Nov 1;31(5):533-8.
Greenfield S, Kaplan SH, Ware JE, Yano EM, Frank HJ. Patients' participation in medical care. Journal of general internal medicine. 1988 Sep;3(5):448-57.

Woodcock RW. Theoretical foundations of the WJ-R measures of cognitive ability. Journal of Psychoeducational Assessment. 1990 Sep;8(3):231-58.
Psychological testing, 6th ed. Citation. Anastasi, A. (1988). Psychological testing (6th ed.). Macmillan Publishing Co, Inc.
Hogarty GE, Anderson CM, Reiss DJ, Kornblith SJ, Greenwald DP, Javna CD, Madonia MJ. Family psychoeducation, social skills training, and maintenance chemotherapy in the aftercare treatment of schizophrenia: I. One-year effects of a controlled study on relapse and expressed emotion. Archives of general psychiatry. 1986 Jul 1;43(7):633-42.
Luepker RV, Rästam L, Hannan PJ, Murray DM, Gray C, Baker WL, Crow R, Jacobs Jr DR, Pirie PL, Mascioli SR, Mittelmark MB. Community education for cardiovascular disease prevention: morbidity and mortality results from the Minnesota Heart Health Program. American Journal of Epidemiology. 1996 Aug 15;144(4):351-62.
McGrew KS, Werder JK, Woodcock RW. Woodcock-Johnson [R]: Technical Manual. DLM; 1991.
**1997 to 2006**
Penn DL, Mueser KT. Research update on the psychosocial treatment of schizophrenia. The American Journal of Psychiatry. 1996 May.
Campbell M, Fitzpatrick R, Haines A, Kinmonth AL, Sandercock P, Spiegelhalter D, Tyrer P. Framework for design and evaluation of complex interventions to improve health. Bmj. 2000 Sep 16;321(7262):694-6.
Parker RM, Baker DW, Williams MV, Nurss JR. The test of functional health literacy in adults. Journal of general internal medicine. 1995 Oct 1;10(10):537-41.
Doak CC, Doak LG, Root JH. Teaching patients with low literacy skills. AJN The American Journal of Nursing. 1996 Dec 1;96(12):16M.
Baker DW, Parker RM, Williams MV, Clark WS. Health literacy and the risk of hospital admission. Journal of general internal medicine. 1998 Dec;13(12):791-8.
US Preventive Services Task Force, United States. Office of Disease Prevention, Health Promotion. Guide to clinical preventive services: report of the US Preventive Services Task Force. US Department of Health and Human Services, Office of Public Health and Science, Office of Disease Prevention and Health Promotion; 1996.
The Health and Personal Social Services Programmes. Department of Health The Government's Expenditure Plans 2001-2002 to 2003-2004 and Main Estimates 2001-2002
Osman LM, Abdalla MI, Beattie JA, Ross SJ, Russell IT, Friend JA, Legge JS, Douglas JG. Reducing hospital admission through computer supported education for asthma patients. Bmj. 1994 Feb 26;308(6928):568-71.
Dixon L, Adams C, Lucksted A. Update on family psychoeducation for schizophrenia. Schizophrenia bulletin. 2000 Jan 1;26(1):5-20.
Campbell MK, DeVellis BM, Strecher VJ, Ammerman AS, DeVellis RF, Sandler RS. Improving dietary behavior: the effectiveness of tailored messages in primary care settings. American journal of public health. 1994 May;84(5):783-7.
Rich MW, Beckham V, Wittenberg C, Leven CL, Freedland KE, Carney RM. A multidisciplinary intervention to prevent the readmission of elderly patients with congestive heart failure. New England Journal of Medicine. 1995 Nov 2;333(18):1190-5.
Gallefoss F, Bakke PS, RSGAARD PK. Quality of life assessment after patient education in a randomized controlled study on asthma and chronic obstructive pulmonary disease. American journal of respiratory and critical care medicine. 1999 Mar 1;159(3):812-7.

Roter DL, Hall JA, Merisca R, Nordstrom B, Cretin D, Svarstad B. Effectiveness of interventions to improve patient compliance: a meta-analysis. Medical care. 1998 Aug 1:1138-61.
Litzelman DK, Slemenda CW, Langefeld CD, Hays LM, Welch MA, Bild DE, Ford ES, Vinicor F. Reduction of lower extremity clinical abnormalities in patients with non-insulin-dependent diabetes mellitus: a randomized, controlled trial. Annals of internal medicine. 1993 Jul 1;119(1):36-41.
**2007 to 2011**
Baker DW. The meaning and the measure of health literacy. Journal of general internal medicine. 2006 Aug;21(8):878-83.
DeWalt DA, Berkman ND, Sheridan S, Lohr KN, Pignone MP. Literacy and health outcomes. Journal of general internal medicine. 2004 Dec;19(12):1228-39.
Barlow J, Wright C, Sheasby J, Turner A, Hainsworth J. Self-management approaches for people with chronic conditions: a review. Patient education and counseling. 2002 Oct 1;48(2):177-87.
Ziebland S, Chapple A, Dumelow C, Evans J, Prinjha S, Rozmovits L. How the internet affects patients' experience of cancer: a qualitative study. Bmj. 2004 Mar 4;328(7439):564.
Davis TC, Wolf MS, Bass III PF, Thompson JA, Tilson HH, Neuberger M, Parker RM. Literacy and misunderstanding prescription drug labels. Annals of internal medicine. 2006 Dec 19;145(12):887-94.
Davis TC, Williams MV, Marin E, Parker RM, Glass J. Health literacy and cancer communication. CA: a cancer journal for clinicians. 2002 May;52(3):134-49.
Lewis D. Computers in patient education. CIN: Computers, Informatics, Nursing. 2003 Mar 1;21(2):88-96.
Scott TL, Gazmararian JA, Williams MV, Baker DW. Health literacy and preventive health care use among Medicare enrollees in a managed care organization. Medical care. 2002 May 1;40(5):395-404.
Berland GK, Elliott MN, Morales LS, Algazy JI, Kravitz RL, Broder MS, Kanouse DE, Muñoz JA, Puyol JA, Lara M, Watkins KE. Health information on the Internet: accessibility, quality, and readability in English and Spanish. jama. 2001 May 23;285(20):2612-21.
Schillinger D, Piette J, Grumbach K, Wang F, Wilson C, Daher C, Leong-Grotz K, Castro C, Bindman AB. Closing the loop: physician communication with diabetic patients who have low health literacy. Archives of internal medicine. 2003 Jan 13;163(1):83-90.
Norris SL, Lau J, Smith SJ, Schmid CH, Engelgau MM. Self-management education for adults with type 2 diabetes: a meta-analysis of the effect on glycemic control. Diabetes care. 2002 Jul 1;25(7):1159-71.
Colom F, Vieta E, Martinez-Aran A, Reinares M, Goikolea JM, Benabarre A, Torrent C, Comes M, Corbella B, Parramon G, Corominas J. A randomized trial on the efficacy of group psychoeducation in the prophylaxis of recurrences in bipolar patients whose disease is in remission. Archives of general psychiatry. 2003 Apr 1;60(4):402-7.
**2012 to 2016**
Berkman ND, Sheridan SL, Donahue KE, Halpern DJ, Crotty K. Low health literacy and health outcomes: an updated systematic review. Annals of internal medicine. 2011 Jul 19;155(2):97-107.
Baker DW, Wolf MS, Feinglass J, Thompson JA, Gazmararian JA, Huang J. Health literacy and mortality among elderly persons. Archives of internal medicine. 2007 Jul 23;167(14):1503-9.
Nutbeam D. The evolving concept of health literacy. Social science & medicine. 2008 Dec 1;67(12):2072-8.

Osborn CY, Weiss BD, Davis TC, Skripkauskas S, Rodrigue C, Bass PF, Wolf MS. Measuring adult literacy in health care: performance of the newest vital sign. American journal of health behavior. 2007 Aug 1;31(1):S36-46.
Weiss BD, Mays MZ, Martz W, Castro KM, DeWalt DA, Pignone MP, Mockbee J, Hale FA. Quick assessment of literacy in primary care: the newest vital sign. The Annals of Family Medicine. 2005 Nov 1;3(6):514-22.
Sørensen K, Van den Broucke S, Fullam J, Doyle G, Pelikan J, Slonska Z, Brand H. Health literacy and public health: a systematic review and integration of definitions and models. BMC public health. 2012 Dec;12(1):1-3.
Jordan JE, Osborne RH, Buchbinder R. Critical appraisal of health literacy indices revealed variable underlying constructs, narrow content and psychometric weaknesses. Journal of clinical epidemiology. 2011 Apr 1;64(4):366-79.
Chew LD, Griffin JM, Partin MR, Noorbaloochi S, Grill JP, Snyder A, Bradley KA, Nugent SM, Baines AD, VanRyn M. Validation of screening questions for limited health literacy in a large VA outpatient population. Journal of general internal medicine. 2008 May 1;23(5):561-6.
DeWalt DA, Hink A. Health literacy and child health outcomes: a systematic review of the literature. Pediatrics. 2009 Nov 1;124(Supplement 3):S265-74.
Bennett IM, Chen J, Soroui JS, White S. The contribution of health literacy to disparities in self-rated health status and preventive health behaviors in older adults. The Annals of Family Medicine. 2009 May 1;7(3):204-11.
Osborn CY, Cavanaugh K, Wallston KA, Kripalani S, Elasy TA, Rothman RL, White RO. Health literacy explains racial disparities in diabetes medication adherence. Journal of health communication. 2011 Sep 30;16(sup3):268-78.
Greene J, Hibbard JH. Why does patient activation matter? An examination of the relationships between patient activation and health-related outcomes. Journal of general internal medicine. 2012 May 1;27(5):520-6.
**2017 to 2021**
Sørensen K, Pelikan JM, Röthlin F, Ganahl K, Slonska Z, Doyle G, Fullam J, Kondilis B, Agrafiotis D, Uiters E, Falcon M. Health literacy in Europe: comparative results of the European health literacy survey (HLS-EU). European journal of public health. 2015 Dec 1;25(6):1053-8.
Diviani N, van den Putte B, Giani S, van Weert JC. Low health literacy and evaluation of online health information: a systematic review of the literature. Journal of medical Internet research. 2015;17(5):e112.
Nguyen HC, Nguyen MH, Do BN, Tran CQ, Nguyen TT, Pham KM, Pham LV, Tran KV, Duong TT, Tran TV, Duong TH. People with suspected COVID-19 symptoms were more likely depressed and had lower health-related quality of life: the potential benefit of health literacy. Journal of clinical medicine. 2020 Apr;9(4):965.
Miller TA. Health literacy and adherence to medical treatment in chronic and acute illness: A meta-analysis. Patient education and counseling. 2016 Jul 1;99(7):1079-86.
Haun JN, Valerio MA, McCormack LA, Sørensen K, Paasche-Orlow MK. Health literacy measurement: an inventory and descriptive summary of 51 instruments. Journal of health communication. 2014 Oct 14;19(sup2):302-33.
Wang C, Pan R, Wan X, Tan Y, Xu L, Ho CS, Ho RC. Immediate psychological responses and associated factors during the initial stage of the 2019 coronavirus disease (COVID-19) epidemic among the general population in China. International journal of environmental research and public health. 2020 Jan;17(5):1729.

Stacey D, Légaré F, Lewis K, Barry MJ, Bennett CL, Eden KB, Holmes-Rovner M, Llewellyn-Thomas H, Lyddiatt A, Thomson R, Trevena L. Decision aids for people facing health treatment or screening decisions. Cochrane database of systematic reviews. 2017(4).
Duong VT, Lin IF, Sorensen K, Pelikan JM, Van den Broucke S, Lin YC, Chang PW. Health literacy in Taiwan: a population-based study. Asia Pacific Journal of Public Health. 2015 Nov;27(8):871-80.
Mackey LM, Doody C, Werner EL, Fullen B. Self-management skills in chronic disease management: what role does health literacy have?. Medical Decision Making. 2016 Aug;36(6):741-59.
Rikard RV, Thompson MS, McKinney J, Beauchamp A. Examining health literacy disparities in the United States: a third look at the National Assessment of Adult Literacy (NAAL). BMC Public Health. 2016 Dec;16(1):1-1.
Altin SV, Finke I, Kautz-Freimuth S, Stock S. The evolution of health literacy assessment tools: a systematic review. BMC public health. 2014 Dec;14(1):1-3.
Hadlaczky G, Hökby S, Mkrtchian A, Carli V, Wasserman D. Mental Health First Aid is an effective public health intervention for improving knowledge, attitudes, and behaviour: A meta-analysis. International Review of Psychiatry. 2014 Aug 1;26(4):467-75.
Brooks SK, Webster RK, Smith LE, Woodland L, Wessely S, Greenberg N, Rubin GJ. The psychological impact of quarantine and how to reduce it: rapid review of the evidence. The lancet. 2020 Mar 14;395(10227):912-20.
World Health Organization. Global report on diabetes: executive summary. World Health Organization; 2016.
International Diabetes Foundation. IDF DIABETES ATLAS. Eighth edition 2017
WHO. WHO declares COVID-19 a pandemic. 2020. https://www.who.int/emergencies/diseases/novel-coronavirus-2019
